# DeepMF: deciphering the latent patterns in omics profiles with a deep learning method

**DOI:** 10.1186/s12859-019-3291-6

**Published:** 2019-12-27

**Authors:** Lingxi Chen, Jiao Xu, Shuai Cheng Li

**Affiliations:** City University of Hong Kong, 83 Tat Chee Ave, Kowloon Tong, Hong Kong, China

**Keywords:** Matrix factorization, Dimension reduction, Deep learning, Omics profile, Cancer subtype

## Abstract

**Background:**

With recent advances in high-throughput technologies, *matrix factorization* techniques are increasingly being utilized for mapping quantitative omics profiling matrix data into low-dimensional embedding space, in the hope of uncovering insights in the underlying biological processes. Nevertheless, current matrix factorization tools fall short in handling noisy data and missing entries, both deficiencies that are often found in real-life data.

**Results:**

Here, we propose DeepMF, a deep neural network-based factorization model. DeepMF disentangles the association between molecular feature-associated and sample-associated latent matrices, and is tolerant to noisy and missing values. It exhibited feasible cancer subtype discovery efficacy on mRNA, miRNA, and protein profiles of medulloblastoma cancer, leukemia cancer, breast cancer, and small-blue-round-cell cancer, achieving the highest clustering accuracy of 76%, 100%, 92%, and 100% respectively. When analyzing data sets with 70% missing entries, DeepMF gave the best recovery capacity with silhouette values of 0.47, 0.6, 0.28, and 0.44, outperforming other state-of-the-art MF tools on the cancer data sets Medulloblastoma, Leukemia, TCGA BRCA, and SRBCT. Its embedding strength as measured by clustering accuracy is 88%, 100%, 84%, and 96% on these data sets, which improves on the current best methods 76%, 100%, 78%, and 87%.

**Conclusion:**

DeepMF demonstrated robust denoising, imputation, and embedding ability. It offers insights to uncover the underlying biological processes such as cancer subtype discovery. Our implementation of DeepMF can be found at https://github.com/paprikachan/DeepMF.

## Background

Recent advances in high-throughput technologies have eased the quantitative profiling of biological data [1]. In many cases, the biological data are captured in a high-dimensional matrix with molecular features such as gene, mutation locus, or species as rows and samples/repetition as columns. Values in the matrices are typically measurements such as expression abundances, mutation levels, or species counts. Based on the assumption that samples with the similar phenotype (or molecular features) that participate in a similar biological process will share similar distribution of biological variation [1], researchers leverage clustering methods like *k*-means and hierarchical clustering to identify similar patterns and discover molecular features or sample subgroups [2, 3]. Nevertheless, these clustering methods might fail to capture the full scape of underlying structures, which may debilitate the accuracy of subgroup identification and introduce bias to the underlying biological process. Thus, in this field, researchers increasingly adopt dimension reduction techniques and utilize the inferred alternative low-dimensional structure as input for subgroups clustering [1, 2].

**Fig. 1:**
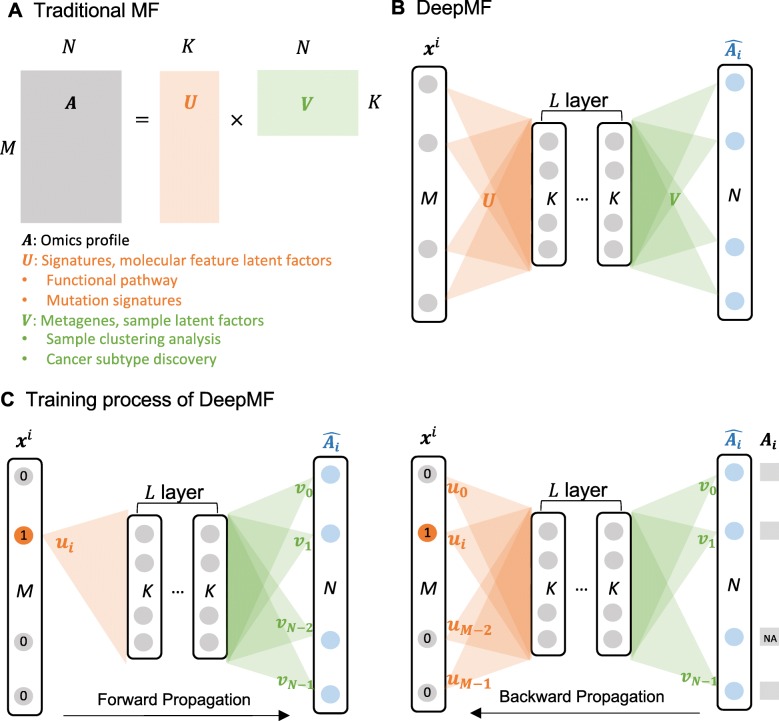
DeepMF structure overview. **a**-**b** Illustration of MF and DeepMF, respectively. **c** The training process of DeepMF. The triangles represent the *K* dimensional molecular feature latent factors $\boldsymbol {u}\in \mathbb {R}^{K}$ or sample latent factors $\boldsymbol {v}\in \mathbb {R}^{K}$

Matrix factorization (MF), as given by the formula $\boldsymbol {A} \in \mathbb {R}^{M \times N} \approx \boldsymbol {U} \in \mathbb {R}^{M \times K} \times \boldsymbol {V} \in \mathbb {R}^{K \times N}$ in Fig. 1a, is a popular approach to infer low-dimensional pattern from high-dimensional omics data [1]. MFs decipher two sets of *K*-dimensional hidden representations from high-dimensional data: one explaining molecular relationship ***U*** and another describing sample-level connection ***V***. We refer ***U*** as the signatures or molecular feature latent matrix, since the values in each column of ***U*** are continuous weights illustrating the relative participation of a molecule in each inferred biology process signature. Leveraging the molecular feature latent matrices learned from gene expression profiles, the data-driven functional pathways can be identified [4–6]. MF has also been used to define COSMIC mutational signatures in pan-cancer studies with patients mutation profiles [7–9]. We call ***V*** the metagenes or sample latent matrix, as each column of ***V*** represents the genes in embedding space and each row of ***V*** depicts the fractions of samples in the matched biological process signature. Patient subgroups discovery is well enabled by analysis of the sample latent matrix. For instance, detecting leukemia cancer subtype based on expression profiles [2], classifying HPV subtypes in head and neck tumors by integrating gene expression and DNA methylation data[10], and The Cancer Genome Atlas (TCGA) pan-cancer patients subtyping from mutation profiles [11].

In biological field, current widely applied MF methods are principal component analysis (PCA), independent component analysis (ICA), and non-negative matrix factorization (NMF). Intuitively, PCA finds governing variation in high-dimensional data, securing the most important biological process signatures that differentiate between samples [12]. ICA separates mixed-signal matrix into statistically independent biological process signatures [13]. NMF-based approaches extracted signatures and metagenes matrices with non-negative constraints [14, 15]. Despite the effectiveness of MF in interpreting biological matrices, several limitations persist in practice. First, real-world data are often plagued with many types of noises, e.g., systematic noise, batch effect, and random noise [16], which potentially mask signals in the downstream process. Second, high throughput omics data frequently suffer from missing values due to various experimental settings [17], whereas the majority of MF tools have no support for input matrix with missing values. At present, the standard practice to deal with these two problems is to perform denoising and imputation prior to MF. In the meantime, deep learning based matrix factorization architectures are developed in the recommendation system field [18, 19]. Those architectures employ two deep encoders to map column and row factors into low-dimensional embedding space, respectively, and apply cosine similarity or multiple layer perceptron as the decoder to refine the existing and predict the missing rating scores.

In this study, we focus on the problem of the cancer subtyping, and propose a novel deep neural network-based matrix factorization framework, DeepMF (see Fig. 1b), which seperately maps molecular features and samples into low-dimensional latent space, tolerant with noisy and missing entries. First of all, we demonstrate DeepMF is robust in denoising, imputation, and embedding with in silico instances. Then, we collected four wet lab datasets, medulloblastoma cancer, leukemia cancer, breast cancer, and small-blue-round-cell cancer datasets, as benchmark sets to evaluate the tools. DeepMF outperformed the existing MF tools on cancer subtype discovery in omics profiles of the four benchmark datasets, with the highest clustering accuracy on all the four datasets. Furthermore, with 70% data randomly removed, DeepMF demonstrated the best recovery capacity with silhouette values 0.47, 0.6, 0.28, and 0.44. It also displayed the best embedding power on the four sparse benchmark sets, with clustering accuracy of respectively 88%, 100%, 84%, and 96%, which improves on the current best methods 76%, 100%, 78%, and 87%.

## Method

### Matrix factorization by deep neural network

In this section, we introduce the DeepMF architecture and the loss function used for its training. Unless stated otherwise, symbols in bold font refer to vectors or matrices.

#### Matrix factorization

In Fig. 1a, assume the input matrix ***A*** is of dimension *M*×*N*, where *M* is the number of features, and *N* is the number of samples. A row represents a feature, while a column represents a sample or a replication. The element ***A***_*ij*_ refers to the measured values for feature *F*^*i*^ on sample *S*^*j*^,0≤*i*≤*M*−1,0≤*j*≤*N*−1.

Matrix factorization assumes the dot product of feature latent factor ***u***^*i*^ and sample latent factor ***v***^*j*^ to capture the interactions between feature *F*^*i*^ and sample *S*^*j*^, where ***u***^*i*^ and ***v***^*j*^ are vectors of size *K* which encode structures that underlie the data; that is, the predicted element of feature *F*^*i*^ on sample *S*^*j*^ is calculated as:

$$\hat{\boldsymbol{A}}_{ij} \approx \sum_{k} \boldsymbol{u}^{i}_{k} \boldsymbol{v}^{j}_{k} = \boldsymbol{u}^{\top i}\boldsymbol{v}^{j} $$

The predicted matrix $\hat {\boldsymbol {A}}$ can be thought of as the product of the feature latent factor matrix ***U*** and sample latent factor matrix $\boldsymbol {V}, \hat {\boldsymbol {A}} \in \mathbb {R}^{M \times N} \approx \boldsymbol {U} \times \boldsymbol {V} $, where $\boldsymbol {U} \in \mathbb {R}^{M \times K}, \boldsymbol {V} \in \mathbb {R}^{K \times N}, K \ll M, N$. The objective function is $ \min _{\boldsymbol {U},\boldsymbol {V}} ||\boldsymbol {A} - \hat {\boldsymbol {A}}||^{2}_{2}$.

#### Framework architecture

Figure 1b illustrates the network architecture of DeepMF. The input layer has *M* neurons, corresponding to *M* features in the matrix. The output layer has *N* nodes to model the *N* column samples. The middle of network is *L* hidden layers of *K* nodes each. All the nodes in the hidden layers are fully connected and paired with ReLU activation function. The number of nodes, *K*, corresponds to the dimensionality of the latent space in matrix factorization. The weights of the first and last layers are respectively considered as the feature latent factors ***U*** and sample latent factors ***V***.

#### Training

Figure 1c reveals the training process of DeepMF. The matrix $\boldsymbol {A} \in \mathbb {R}^{M \times N}$ contains *M* features. Each feature *F*^*i*^ corresponds to one input data point ***x***^*i*^∈[0,1]^*M*^ and output label $\boldsymbol {y}^{i} \in \mathbb {R}^{N} $, where ***x***^*i*^ is one-hot encoded and ***y***^*i*^ is the *i*-th row of matrix ***A***.

$$\begin{array}{*{20}l} \boldsymbol{x}^{i} &= \left[\overbrace{0... 0 \underbrace{1}_{i\texttt{-th feature, \(F^{i}\)}} 0... 0}^{M}\right] \\ \boldsymbol{y}^{i} &= \boldsymbol{A}_{i} \end{array} $$

The loss function consists of two parts, one for global trends and one for local trends. For a pair of feature *F*^*i*^ and sample *S*^*j*^,*global* *proximity* refers to the proximity between real measurement ***A***_*ij*_ and predicted value $\hat {\boldsymbol {A}}_{ij}$. The preservation of global proximity is fundamental in matrix factorization. On the other hand, if two samples possess many common features, they tend to be similar. We refer to this similarity as *sample* *local* *proximity*. We define *feature* *local* *proximity* similarly in the same way. By introducing these local proximities into the loss function, we aim to identify and preserve the sample-pairwise and feature-pairwise structures in the low-dimensional latent space.

For global proximity, we minimize the $\mathcal {L}$2-norm of the residual:

1 $$\begin{array}{*{20}l} \mathcal{L}_{global} &= \frac{1}{M} \sum_{i=1}^{M} || \boldsymbol{y}^{i} - \hat{\boldsymbol{y}}^{i} ||^{2}_{2} \end{array} $$

For the local proximities, we use feature local proximity $\boldsymbol {S}^{F}_{M \times M}$ and sample local proximity $\boldsymbol {S}^{S}_{N \times N}$ as supervised information. Given matrix ***A***_*M*×*N*_, we obtain the feature similarity matrix $\boldsymbol {S}^{F}_{M \times M}$ and sample similarity matrix $\boldsymbol {S}^{S}_{N \times N}$ as following.

2 $$\begin{array}{*{20}l} s^{F}_{kl} &= \frac{1}{1 + || \boldsymbol{A}_{k} - \boldsymbol{A}_{l} ||^{2}_{2}} \end{array} $$

3 $$\begin{array}{*{20}l} s^{S}_{kl} &= \frac{1}{1 + || \boldsymbol{A}^{\top}_{k} - \boldsymbol{A}^{\top}_{l}||^{2}_{2}} \end{array} $$

where ***A***_*k*_ and ***A***_*l*_ refer to the *k*-th and *l*-th row of matrix ***A***. $\boldsymbol {A}^{\top }_{k}$ and $\boldsymbol {A}^{\top }_{l}$ refer to the *k*-th and *l*-th column of matrix ***A***.

To preserve the local proximity, we use ***S***^*F*^ and ***S***^*S*^ respectively constrain the similarity of the latent representations of features ***U*** and samples ***V***.

4 $$\begin{array}{*{20}l} \mathcal{L}_{local} &= \sum_{k,l=1}^{M} s^{F}_{kl} || \boldsymbol{U}_{k} - \boldsymbol{U}_{l} ||^{2}_{2} \\ &+ \sum_{k,l=1}^{N} s^{S}_{kl} || \boldsymbol{V}^{\top}_{k} - \boldsymbol{V}^{\top}_{l} ||^{2}_{2} \\ & = 2\text{trace}\left(\boldsymbol{U}^{\top}\boldsymbol{L}^{F}\boldsymbol{U}\right) + 2\text{trace}\left(\boldsymbol{V}\boldsymbol{L}^{S}\boldsymbol{V}^{\top}\right) \end{array} $$

where ***U***_*k*_ and ***U***_*l*_ refer to the *k*-th and *l*-th row of feature latent matrix ***U***. $\boldsymbol {V}^{\top }_{k}$ and $\boldsymbol {V}^{\top }_{l}$ refer to the *k*-th and *l*-th column of sample latent matrix ***V***. ***L***^*F*^=***D***^*F*^−***S***^*F*^ and ***L***^*S*^=***D***^*S*^−***S***^*S*^ are the Laplacian matrices for features and samples, respectively. $\boldsymbol {D}^{F} \in \mathbb {R}^{M \times M}$ and $ \boldsymbol {D}^{S} \in \mathbb {R}^{N \times N}$ are diagonal matrices with $d^{F}_{kk} = \sum _{l}s^{F}_{kl}$ and $d^{S}_{kk} = \sum _{l}s^{S}_{kl}$.

The objective function $\mathcal {L}_{local}$ incurs a graph Laplacian penalty when similar features and similar samples are embedded far away in the latent space. Hence, two features or samples with low similarity will be driven nearer in the embedding space. To prevent this, we first identify the remote sample-sample or feature-feature pair from feature and sample local proximity matrices by *k*-means. Then we mark their local similarity to zero to exclude them from $\mathcal {L}_{local}$ constraints.

To avoid overfitting and constrain the latent matrices ***U*** and ***V***, an $\mathcal {L}$2-norm regularization is incorporated with ***U***,***V***, and model hidden layer weights ***W***_*hidden*_.

5 $$\begin{array}{*{20}l} \mathcal{L}_{reg} = ||\boldsymbol{U}||^{2}_{2} + ||\boldsymbol{V}||^{2}_{2} + ||\boldsymbol{W}_{hidden}||^{2}_{2} \end{array} $$

Our final loss function incorporates all the above constraints, with two additional hyperparameters *α* and *β*, as follows:

6 $$\begin{array}{*{20}l} \mathcal{L}_{mix} = \mathcal{L}_{global} + \alpha\mathcal{L}_{local} + \beta\mathcal{L}_{reg}. \end{array} $$

#### Dealing with missing value

To be tolerant of missing values, DeepMF discards the missing entries in back-propagation by a variational $\mathcal {L}$2-norm (see Fig. 1c). Denote *ξ* as a missing value.

7 $$\begin{array}{*{20}l} \mathcal{L}_{global}^{missing} &= \frac{1}{M} \sum_{i=1}^{M}\sum_{j=1}^{N} loss^{ij} \\ loss^{ij} &= \left\{ \begin{array}{ll} 0, \boldsymbol{y}^{i}_{j}=\xi, \\ || \boldsymbol{y}^{i}_{j} - \hat{\boldsymbol{y}}^{i}_{j} ||^{2}_{2}, \boldsymbol{y}^{i}_{j}\ne\xi. \end{array} \right. \end{array} $$

Then, DeepMF can infer a missing value of ***A***_*αβ*_ by utilizing the trained model.

$$\begin{array}{*{20}l} \boldsymbol{x}^{\alpha} &= [\overbrace{0... 0 \underbrace{1}_{\alpha\texttt{-th feature, \(F^{\alpha}\)}} 0... 0}^{M}]\\ \hat{\boldsymbol{y}}^{\alpha} &= \texttt{DeepMF.predict}(\boldsymbol{x}^{\alpha}) \\ \hat{\boldsymbol{A}}_{\alpha\beta} &= \hat{\boldsymbol{y}}^{\alpha}_{\beta}. \end{array} $$

#### DeepMF architecture parameter selection

If the data assumes *C* (*C*≥2) clusters with respect to samples, we recommend that the network structure be pruned as guided by the validation loss $\mathcal {L}_{mix}$ in the range of *K*∈[2,*C*] and *L*∈[1,+*∞*). For a matrix ***V***_*K*×*N*,(*K*<*N*)_, a rank of *C* is enough to represent the latent hierarchical structure for a *C*-clustering problem, thus *K*≤*C*. To extract simple patterns between feature and sample, *L*=1 suffices. A larger *L* would provide more complexity in the latent space of DeepMF. For hyperparameter tuning, we recommend running each *K,L* combination more than ten times with different random weights initialization to avoid possible local optima.

### Simulation data generation

**Fig. 2:**
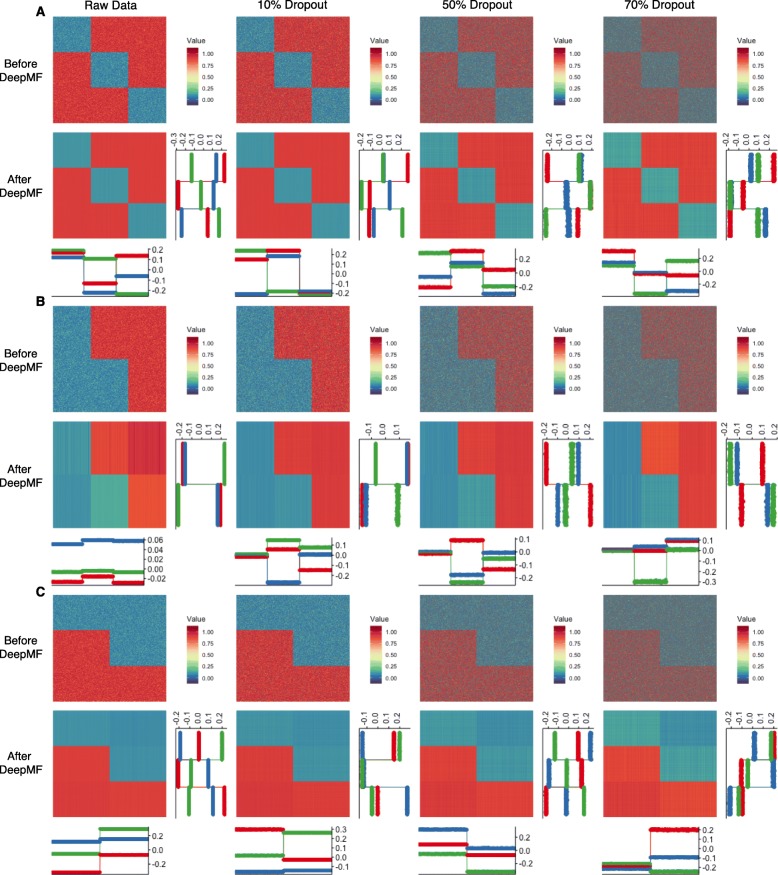
DeepMF performance on 1000×600 synthetic matrices. DeepMF denoising, imputation, and factorization performance on 1000×600 synthetic matrices with different pattern. Inside each pattern, from left to right: raw matrix, 10% random dropout, 50% random dropout, 70% random dropout; from top to bottom: before DeepMF, and DeepMF. The horizontal line plot show the sample latent factors, the vertical line plot refer to feature latent factors. **a** Matrix with pattern A **b** Matrix with pattern B **c** The transpose matrix of pattern B

To evaluate DeepMF, we simulated three patterns, each which consists of matrices of sizes 1000×600,10×6, and 100×60 as shown in Fig. 2, Additional files 1 and 2. Then, we randomly removed 10%, 50%, and 70% of the matrices to make them sparse.

### Cancer subtyping experiments

For real datasets, the four cancer datasets as follows are used.

#### Cancer data preparation

**Medulloblastoma data set** Gene expression profiles from childhood brain tumors medulloblastomas were obtained from Brunet’s work [2]. It consists of classic and desmoplastic subtypes of size 25 and 9, respectively. We further extracted the top 100 differentially expressed genes using the “limma" R package [20].

**Leukemia data set** The Leukemia data set was obtained from R package “NMF” with the command “data(esGolub)” [14]. It stores Affymetrix Hgu6800 microarray expression data from 38 Leukemia cancer patients, where 19 patients with B cell Acute Lymphoblastic Leukemia (B-cell ALL), eight patients with T cell Acute Lymphoblastic Leukemia (T-cell ALL), as well as 11 patients with Acute Myelogenous Leukemia (AML). The 236 most highly diverging genes were selected by comparison on their coefficient of variation using the “limma” R package [20].

**TCGA BRCA data set** A subset of human breast cancer data generated by The Cancer Genome Atlas Network (TCGA) was obtained from R package mixOmics [21]. It holds 150 samples with three subtypes Basal-like, Her2, and LumA, of size 45, 30, and 75, respectively. The top 55 correlated mRNA, miRNA, and proteins that discriminate against the breast cancer subgroups Basal, Her2, and LumA were selected using the mixOmics DIABLO model.

**SRBCT data set** The Small Round Blue Cell Tumors (SRBCT) data set holds the expression profiles of the top 96 ranked genes [22]. It contains 63 samples of four classes, Burkitt Lymphoma (BL, eight samples), Ewing Sarcoma (EWS, 23 samples), Neuroblastoma (NB, 12 samples), and Rhabdomyosarcoma (RMS, 20 samples). The processed and normalized data were acquired from the R mixOmics package [21].

#### Decomposition baselines

We compared the decomposition efficacy on DeepMF against four methods, PCA (FactoMineR [23]), ICA (fastICA[24]), Bayesian-based NMF (CoGAPS[15]), and gradient-based NMF (NMF [14]). We fit all model with log-treated matrices. All tools were executed with their recommended settings; that is, prcomp function in package “FactoMineR”; fastICA with algorithm type “parallel”, function “logcosh”, alpha 1, method “R”, row normalization 1, maxit 200, tol 0.0001; CoGAPS with 5000 iterations; NMF with method “brunet" and 200 runs.

As CoGAPS and NMF accept only non-negative values, we used NMF.posneg to transform the input matrices into corresponding non-negative matrices.

#### Imputation baselines

We evaluated the DeepMF imputation efficiency by comparing it with two popular imputation approaches, MeanImpute, and SVDImpute.

**MeanImpute** MeanImpute adopted the approach that the missing entries are to be substituted by the mean of the current values of a particular feature in all samples. We used the mean impute function in the R package “CancerSubtypes”.

**SVDImpute** SVDImpute first centers the matrix, replaces all missing values by 0, decomposes the matrix into the eigenvectors. Then, SVDImpute predicts the NA values as a linear combination of the *k* most significant eigenvectors [25]. We chose SVDImpute as an imputation baseline since the mechanism behind it is similar to DeepMF. The *k* most significant eigenvectors can be analogized to the *k*-dimensional latent matrix in DeepMF. We used R package “pcaMethods” in practice.

#### Deep matrix factorization model baseline

There are some deep learning-basedd matrix factorization architectures in the recommendation system field [18, 19]. Given a rating matrix for pairs of user and item, those architectures are designed to respectively map users and items into low-dimensional embedding space, refine the existing and predict the missing rating scores in the meantime. Nevertheless, we fail to find the available source codes. To evaluate the imputing, denoising, and embedding of those deep learning matrix factorization architectures in biological omics data, we implement them using PyTorch and refer it as DMF model in this study. DMF employs two encoders to separately obtain the feature latent factor ***u***^*i*^ and sample latent factor ***v***^*j*^ into low-dimensional embedding space. Then, DMF concatenate $\boldsymbol {u}^{i} \in \mathbb {R}^{K}$ and $\boldsymbol {v}^{j} \in \mathbb {R}^{K}$ into $\boldsymbol {z}^{ij} \in \mathbb {R}^{2K}$, fit ***z***^*ij*^ into a multiple layer perceptron to get the predicted value $\hat {\boldsymbol {A}}_{ij}$. The loss function is binary cross-entropy with sigmoid activation.

#### Evaluation metrics

**Silhouette width** The silhouette width measures the similarity of a sample to its class compared to other classes [26]. It ranges from -1 to 1. A higher silhouette value implies a more appropriate clustering. A silhouette value near 0 intimates overlapping clusters, and a negative value indicates that the clustering has been performed incorrectly.

We adopted the silhouette width to evaluate the model’s denoising and imputation power. We used the ground-truth subtype classes as the input cluster labels. Then, the silhouette width for a given matrix was calculated with Euclidean distance using the R package “cluster”.

**Adjusted Rand Index** We also used the adjusted Rand index to evaluate the clustering accuracy. The adjusted Rand index measures the similarity between predicted clustering results and actual clustering labels [27]. A negative value or value close to 0 indicates random labeling, and a value of 1 demonstrates 100% accuracy of clustering.

To check the cancer subtyping effectiveness of different matrix factorization tools. We first used the R hierarchy clustering packaging “hclust” to obtain the sample latent factor matrices in order to partition samples into subgroups, through the Euclidean distance and “ward.D2" linkage. Then, we computed the adjusted Rand index to measure the clustering accuracy via the R package “fpc”.

## Results

Given matrix $\boldsymbol {A} \in \mathbb {R}^{M \times N}$, DeepMF operates matrix factorization on the basis of deep neural network, outputs three matrices $\boldsymbol {U} \in \mathbb {R}^{M \times K}, \boldsymbol {V} \in \mathbb {R}^{K \times N}$, and $\hat {\boldsymbol {A}} \in \mathbb {R}^{M \times N}$ (see Fig. 1). ***U*** is the weights of the first layer, we considered it as the low-dimensional feature latent factors. The weights of the last layer ***V*** is the sample latent factors in embedding space. Hence we can apply ***U*** and ***V*** to features and samples related clustering and subgroups identification. DeepMF learns about missing values and minimizes the loss between ***A*** and $\hat {\boldsymbol {A}}$ during training, $\hat {\boldsymbol {A}}$ is the refined matrix with no missing and noisy entries.

### Denoising, imputation, and embedding evaluation on synthetic data

To evaluate the denoising, imputation, and embedding efficacy of DeepMF, we first generated three simple patterns A, B, and C, each which consists of matrices of size 1000×600,10×6, and 100×60 (see Fig. 2, Additional files 1 and 2). Matrices with pattern A hold three subgroups in feature and sample. Pattern B has two subgroups in feature and three subgroups in sample. Pattern C matrices are transposed of pattern B of dimension 600×1000,6×10, and 60×100. Then we generated sparse matrices randomly by dropping the entries of matrices with rate 10%, 50%, and 70%.

Figure 2, Additional files 1 and 2 show the performance of DeepMF on the raw matrix and sparse matrix with size 1000×600,10×6, and 100×60, respectively. In Fig. 2a, Additional files 1A and 2A, the DeepMF predicted matrices significantly reduced the noisy and missing entries. In spite of the noise and 70% missing entries, the feature latent factors and sample latent factors generated by DeepMF consistently uncovered ground truth feature subgroups and sample subgroups with 100% accuracy. The same conclusion applies to pattern B and pattern C (see Fig. 2b-c, Additional files 1B-C and 2B-C). We note that pattern B matrices and pattern C matrices are transposed, which suggests that DeepMF can uncover the feature and sample subclasses either from a feature-sample matrix or its transposed matrix. Since fitting a matrix with *N*<*M* is more efficient than a matrix with *N*>*M* in DeepMF, it is unnecessary to adhere to the paradigm of “treating the feature as row and sample as column” [1].

### DeepMF accurately elucidates cancer subtypes on multiple cancer omics data sets

Then, we demonstrate the use of DeepMF in the problem of clarifying cancer subtypes. We collected four cancer omics data sets as benchmark, namely the Medulloblastoma data set (mRNA) [2], Leukemia data set(mRNA) [2, 14], TCGA BRCA data set (mRNA, miRNA, protein) [21], and small blue round cell tumor (SRBCT) data set (mRNA) [21, 22]. Firstly, we verified the denoising power of DeepMF compared with deep learning based MF (DMF [18, 19]), by utilizing the silhouette validation to corroborate whether the in-cluster similarity and out-cluster separation were enhanced after processing. Secondly, incorporating hierarchy clustering, we compared the decomposition efficacy on DeepMF against five baseline methods: PCA (FactoMineR [23]), ICA (fastICA [24]), Bayesian based NMF (CoGAPS [15]), gradient based NMF(NMF [14]), and DMF. Clustering accuracy is evaluated by the adjusted Rand index, which measures the overlap between the inferred clusters and ground-truth subtypes, negative score, or a score close to 0 signifies random labeling, and 1 denotes perfect inference.

**Fig. 3:**
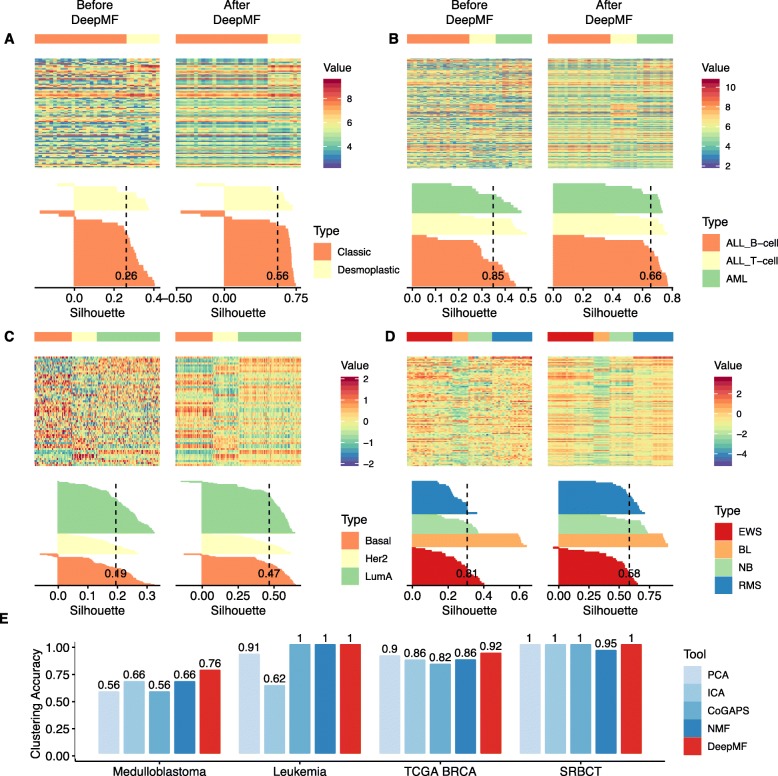
DeepMF denoising and factorization on cancer data sets. **a-d** The heatmap presentation and Silhouette width of four cancer data sets. Left: before DeepMF. Right: after DeepMF. **a** Medulloblastoma data set **b** Leukemia data set **c** TCGA BRCA data set **d** SRBCT data set **e** Clustering accuracy of cancer subtyping on sample latent matrices generated by five matrix factorization tools on different cancer data sets

We first analyzed the benchmark dataset, Medulloblastoma dataset, used in Brunet’s paper to evaluate the gradient-descent NMF tool [2]. Medulloblastomas are childhood brain tumors, and consist of two generally accepted histological subtypes: classic and desmoplastic. We applied PCA, ICA, Bayesian based NMF, gradient based NMF, DMF, and DeepMF to the expression profiles of 34 Medulloblastoma patients with rank *K*=2. The DeepMF structure configuration in training is listed in Additional file 3. To escape from local optima caused by DeepMF random weight initialization, we conducted ten different runs and selected the latent matrices with the model selection criteria defined in Method, that is, we chose the minimum loss $\mathcal {L}_{mix}$. We first verified the correctness of the refined matrices. Figure 3a shows that DeepMF diminished the noise in raw matrices while preserving cancer subtype structures. Silhouette validation corroborated that the in-cluster similarity and out-cluster separation were enhanced after DeepMF processing; that is, the average silhouette value was increased from 0.26 to 0.56. While after multiple tries, DMF can only produce a faulty output with a silhouette value of 0 (see Additional file 4A).

Then, we fitted the obtained sample latent matrices into hierarchical clustering. Figure 3e and Additional file 5 demonstrate that DeepMF outperforms five baseline methods with the highest clustering accuracy of 76%. Additional files 4A, 6A, and 7A illustrate the hierarchical structures and clustering results of the obtained sample latent matrices. All methods consistently misclassify two samples, the classic Brain_MD_49 and desmoplastic Brain_MD_28. Possible explanations might be the incorrect diagnosis of the samples. If we treat them as outliers, then DeepMF correctly distinguished the remaining patients. However, ICA and NMF still incorrectly assign one classic patient Brain_MD_1. PCA and CoGAPs still misclassify two classic patients, Brain_MD_1 and Brain_MD_5. DMF yields a sample latent matrix with no distinction between classic and desmoplastic subgroups, thus fail to identify any sample subtype.

We next employed the six tools to a classic cancer subtyping dataset Golub Leukemia Data Set [2, 14]. It has 38 bone marrow samples consisting of three subgroups, 19 B-cell acute lymphoblastic leukemia (ALL), 8 T-cell ALL, and 11 acute myelogenous leukemia (AML). Thus, rank *K*=3 was selected for all six tools. DeepMF was trained in ten different runs with structure configuration listed in Additional file 3, result with minimum model selection criteria $\mathcal {L}_{mix}$ was selected for evaluation and analysis. We first verified the correctness of the output matrices. Figure 3b shows that DeepMF reduces the noise in the raw matrix while preserving the three leukemia cancer subtype structures. After DeepMF processing, the average silhouette value was increased from 0.35 to 0.66. While DMF masks all subtype-specific signals, yields a silhouette value of -0.1 (see Additional file 4B). Then, we checked whether the DeepMF produced sample latent matrix preserves the cancer subtype information. We applied hierarchical clustering into obtained sample latent matrix (see Additional file 6B). The sample latent matrices derived from DeepMF, Bayesian based NMF, and gradient based NMF generate compact latent structures, thus leading to 100% hierarchical clustering accuracy (see Fig. 3e and Additional file 6B). While PCA and ICA generate sample latent matrices with looser structures among ground-truth labels, leading to one misclassification (ALL_14749_B’cell) and five misclassifications (ALL_14749_B’cell, ALL_21302_B’cell, ALL_18239_B’cell, ALL_R23_B’cell, AML_6), respectively (see Additional file 7B). There are also no subtype-specific signals in sample latent matrix produced by DMF; thus hierarchical clustering shatters B-cell ALL, T-cell ALL, and AML samples into different clusters (see Additional file 4B).

We then collected a subset of human breast cancer (BRCA) data generated by The Cancer Genome Atlas Network (TCGA). It holds 150 samples with three subtypes Basal-like, Her2, and LumA, of size 45, 30, and 75, respectively. It is an omics profile containing the most varying mRNA, miRNA, and proteins, which together discriminate the breast cancer subtypes. The analysis process and evaluation metrics are the same as the previous two benchmarks, except we set the rank as the number of BRCA subtypes, *K*=3. Firstly, DeepMF reduced the noise in the raw matrix and yielded a compact output, with the average silhouette width was increased from 0.19 to 0.47 (see Fig. 3c). Secondly, DeepMF outperformed all baselines and manifested the best embedding strength, with the highest clustering accuracy of 92% (see Fig. 3e, Additional files 4C and 6C). In Additional file 7C, within 150 patients, only five patients were misclassified (A143, A131, A0E0, A12T, A0RH). CoGAPS displayed ten misclassifications. PCA, ICA, gradient based NMF shared similar subtype assignment, and wrongly attributed subtype to six, eight, eight patients, respectively. In terms of DMF, DMF revealed better denoising ability with a silhouette value of 0.52, while it displayed the worse embedding potency with clustering accuracy of 29% (see Additional files 4C and 5).

The last benchmark data set is the small round blue cell tumors (SRBCT) data set, which holds the expression profiles of the top 96 ranked genes [22]. It contains 63 samples of four classes, Burkitt Lymphoma (BL), Ewing Sarcoma (EWS), Neuroblastoma (NB), and Rhabdomyosarcoma (RMS). Thus, we set the rank as the number of subtypes, *K*=4. The analysis process and evaluation metrics are the same as the previous benchmarks. From the perspective of denoising ability, DMF and DeepMF enhanced average silhouette width from 0.31 to 0.45 and 0.58, respectively (see Fig. 3d and Additional file 4D). In terms of embedding strength, PCA, ICA, CoGAPS, and DeepMF perfectly assign samples to their ground-truth subtypes with 100% accuracy (see Fig. 3e, Additional files 6D and 7D), while Gradient based NMF improperly attributed one patient EWS.T13 to RMS category. DMF successfully identified all BL samples, while the latent representation of the other three subtypes are homologous, leads to the worse clustering accuracy of 59% (see Additional files 4D and 5).

### DeepMF captures the cancer subtype patterns despite 70% random dropouts

Several studies have suggested that missing values in large-scale omics data can drastically obstruct the interpretation of unsupervised cancer subtyping [28]. At present, this is most commonly treated by imputing the missing values before performing the downstream dimension reduction and subtype clustering. To tackle this, DeepMF provides a two-pronged solution by assigning predicted values into missing entries and conducting low-dimensional embedding simultaneously.

**Fig. 4:**
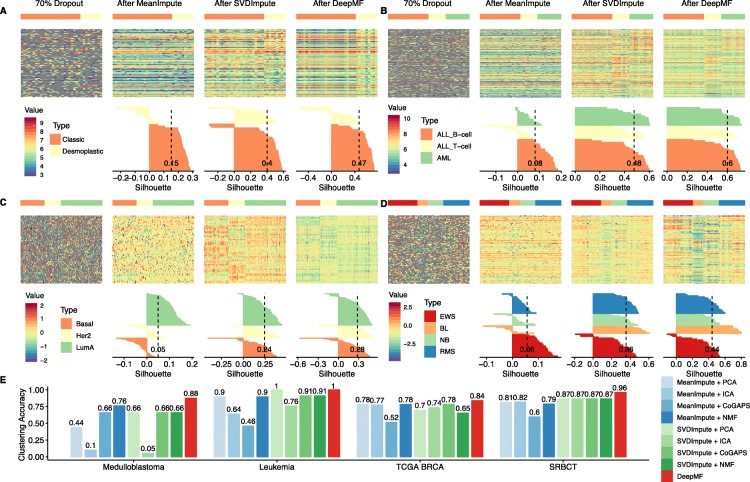
DeepMF’s imputation and factorization effect on cancer data sets with 70% random dropout. **a-d** The heatmap presentation and Silhouette width of four cancer data sets with 70% random dropout. The gray tiles in heatmap indicate missing entries. From left to right: matrix with 70% random dropout, after mean impute, after SVDImpute, after DeepMF. **a** Medulloblastoma data set **b** Leukemia data set **c** TCGA BRCA data set **d** SRBCT data set **e** Clustering accuracy of cancer subtyping on sample latent matrices generated by two imputations and five matrix factorization tools on different cancer data sets with 70% random dropout

To evaluate the efficacy of DeepMF with missing entries, we generate four sparse datasets by randomly discarding 70% entries of the four benchmark data sets. Then we fit the sparse matrices into DMF, DeepMF, and two imputation baselines: MeanImpute and SVDImpute. We selected MeanImpute by considering its popularity. From the perspective of the imputation mechanism, we can regard SVDImpute as a linear analogy of DeepMF. We conducted ten different runs for each data set configuration (see Additional file 3) and picked the one with minimal module selection criteria $\mathcal {L}_{mix}$. Fig. 4 demonstrates that for all 70% missing rate data sets, both DeepMF and SVDImpute recovered distinctive cancer subtype structures, while the MeanImpute approach was unable to reconstruct a clearly visible pattern. Silhouette validation confirmed that DeepMF reduced the most substantial interior cluster heterogeneity and out-cluster similarity, with the largest average silhouette value of 0.47 for the Medulloblastoma data set, 0.6 for the Leukemia data set, 0.28 for TCGA BRCA data set, and 0.44 for SRBCT data set. DMF conducted unsatisfactory imputation jobs, produced negative or close to 0 silhouette values on all sparse benchmark sets (see Additional file 8).

Remainder that we can uncover the cancer subtypes utilizing the sample latent matrices produced by DeepMF. To investigate whether missing entries will hinder DeepMF’s ability in cancer subtyping, we applied hierarchical clustering into sample latent matrices generated by sparse matrices (see Additional file 9) and computed the clustering accuracy with ground-truth subtyping labels (see Fig. 4e). Since the four traditional baseline matrix factorization tools do not accept input with missing values, we fitted the high dimensional matrices treated by MeanImpute and SVDImpute into four baseline approaches. Then we obtained the corresponding low-dimensional sample latent matrices with rank *K*=2 for Medulloblastoma data set, rank *K*=3 for Leukemia data set, rank *K*=3 for TCGA BRCA data set, and rank *K*=4 for SRBCT data set, respectively. Figure 4e shows that in terms of clustering accuracy, DeepMF outperforms all eight imputation and factorization combinations, exhibiting the best embedding power with clustering accuracy of 88% for Medulloblastoma data set, 100% for TCGA BRCA data set, 84% for Leukemia, and 96% for SRBCT data sets. For Medulloblastoma sparse data, DeepMF only incorrectly assigned one desmoplastic sample Brain_MD_28 to classic category, the other eight imputation and MF combinations produced misclassifications range from two to twelve (see Additional files 9A and 10A). In spite of 70% sparsity, SVDImpute + PCA and DeepMF correctly attach each leukemia sample to its right subtype, the other seven baseline tools combinations misclassify leukemia patients range from one to ten (see Additional files 9B and 10B). For 150 TCGA BRCA samples, after removing 70% entries, the clustering accuracy of all tools declined dramatically. DeepMF clustering errors increased from five to nine, the other eight baseline tools combinations produced misclassifications range from 12 to 29 (see Additional files 9C and 10C). In terms of SRBCT data with 70% sparsity, except sample NB.C7, DeepMF correctly attaches each sample to its right subtype. Additional files 9D and 10D illustrate the number of misclassification range from three to eleven for baseline tool combinations. Overall, the clustering results vary on different imputation and MF combinations among different sparse benchmark sets, while DeepMF always demonstrates the best embedding ability with the highest clustering accuracy. Concerning DMF, the sample latent representation among cancer subtypes are not distinguishable, leads to the worse clustering accuracy on all sparse benchmark sets (see Additional files 5 and 8).

## Discussion

In this study, we presented DeepMF, a supervised learning approach to the dimension reduction problem. Unlike current approaches, the method is designed to have high tolerance with respect to noisy data and missing values. Experiments using synthetic and real data corroborated this fact, showing DeepMF to be particularly suited for cancer subtype discovery on omics data, and beats all state of the art MF tools on imputation, denoising, and embedding.

We have not addressed several issues. The first is with regard to the choice of the three hyper-parameters *K,L*,*W* in DeepMF. The choice of the reduced dimensionality *K* is arguably difficult since it is an open problem for the entire dimension reduction research community. A larger *L* would provide more complexity in the latent space of DeepML. To extract simple pattern between feature and sample, *L*=1 suffices. As discussed in methods, if the samples assume *C* (*C*≥2) cancer subtypes, we may search the optimal structure from *K*∈[2,*C*] and *L*∈[1,+*∞*). To find the optimal network structure for accurate cancer subtyping, we defined $\mathcal {L}_{mix}$ to guide the hyperparameter search. Otherwise, we resort to multiple trials for the tuning of these parameters. Even though different combinations of *K,L* might lead to disparate molecular feature and sample latent matrices, all latent matrices enable to preserve the underlying structures of cancer samples as we imposed the graph Laplacian penalty during training.

In this study, we only adopted DeepMF on mRNA, miRNA, and protein data for cancer subtype identification. However, DeepMF is not limited to these data modality and this clustering problem. Human metabolome profiles can undoubtedly benefit from analysis using DeepMF, since the data is known to often suffer from missing values. We intend to apply DeepMF to metabolome and discover signatures beneficial to human health. Furthermore, we plan to employ molecular feature latent matrix to uncover functional pathways in future work.

## Conclusion

MF-based analyses are commonly used in the interpretation of high-throughput biological data. Our proposed DeepMF is an MF-based deep learning framework that overcomes traditional shortcomings such as noise and missing data. Our experiments on simulation data and four omics cancer data sets established DeepMF’s feasibility in denoising, imputation, and in discovering the underlying structure of data.

## Supplementary information

**Additional file 1** DeepMF performance on 10×6 synthetic matrices. DeepMF denoising, imputation, and factorization performance on 10×6 synthetic matrices with different pattern. Inside each pattern, from left to right: raw matrix, 10% random dropout, 50% random dropout, 70% random dropout; from top to bottom: before DeepMF, and DeepMF. The horizontal line plot show the sample latent factors, the vertical line plot refer to feature latent factors. **A** Matrix with pattern A; **B** Matrix with pattern B; **C** The transpose matrix of pattern B.

**Additional file 2** DeepMF performance on 1000×600 synthetic matrices. DeepMF denoising, imputation, and factorization performance on 1000×600 synthetic matrices with different pattern. Inside each pattern, from left to right: raw matrix, 10% random dropout, 50% random dropout, 70% random dropout; from top to bottom: before DeepMF, and DeepMF. The horizontal line plot show the sample latent factors, the vertical line plot refer to feature latent factors. **A** Matrix with pattern A; **B** Matrix with pattern B; **C** The transpose matrix of pattern B.

**Additional file 3** DeepMF training configuration on cancer data sets.

**Additional file 4** DMF denoising and factorization results on cancer data sets. **A-D** The heatmap presentation and Silhouette width of four cancer data sets. From left to right: matrix with before DMF, after DMF. The bottom: hierarchical clustering plots for sample latent matrice generated by DMF. **A** Medulloblastoma data set; **B** Leukemia data set; **C** TCGA BRCA data set; **D** SRBCT data set.

**Additional file 5** DMF adjusted rand index on cancer data sets.

**Additional file 6** Hierarchical clustering plots for sample latent matrices. Sample latent matrices are generated by five matrix factorization tools on different cancer data sets. From top to bottom, each row represents sample latent matrices generated by PCA, ICA, CoGAPS, NMF, DeepMF. **A** Medulloblastoma data set; **B** Leukemia data set; **C** TCGA BRCA data set; **D** SRBCT data set.

**Additional file 7** Hierarchical clustering results for sample latent matrices. The top row is the ground truth subtype label for each patients. The rest rows represent patient subtype assigned by PCA, ICA, CoGAPS, NMF, DeepMF, respectively. **A** Medulloblastoma data set; **B** Leukemia data set; **C** TCGA BRCA data set; **D** SRBCT data set.

**Additional file 8** DMF imputation and factorization results on 70% sparse cancer data sets. **A-D** The heatmap presentation and Silhouette width of four cancer data sets with 70% random dropout. The gray tiles in heatmap indicate missing entries. From left to right: matrix with 70% random dropout, after DMF. The bottom: hierarchical clustering plots for sample latent matrice generated by DMF. **A** Medulloblastoma data set; **B** Leukemia data set; **C** TCGA BRCA data set; **D** SRBCT data set.

**Additional file 9** Hierarchical clustering plots for sample latent matrices generated from 70% random dropout data sets. Sample latent matrices are generated by two imputation tools and five matrix factorization tools on different cancer data sets with 70% random dropout. From top to bottom, each row represents sample latent matrices generated by meanImpute + PCA, meanImpute + ICA, meanImpute + CoGAPS, meanImpute + NMF, SVDImpute + PCA, SVDImpute + ICA, SVDImpute + CoGAPS, SVDImpute + NMF, DeepMF. **A** Medulloblastoma data set; **B** Leukemia data set; **C** TCGA BRCA data set; **D** SRBCT data set.

**Additional file 10** Hierarchical clustering results for sample latent matrices generated from 70% random dropout data sets. The top row is the ground truth subtype label for each patients. The rest rows represent patient subtype assigned by meanImpute + PCA, meanImpute + ICA, meanImpute + CoGAPS, meanImpute + NMF, SVDImpute + PCA, SVDImpute + ICA, SVDImpute + CoGAPS, SVDImpute + NMF, DeepMF, respectively. **A** Medulloblastoma data set; **B** Leukemia data set; **C** TCGA BRCA data set; **D** SRBCT data set.

## Data Availability

The data and source code included in this study can be found in https://github.com/paprikachan/DeepMF.

## Abbreviations

ALL: Acute lymphoblastic leukemia
AML: Acute myelogenous leukemia
BL: Burkitt lymphoma
BRCA: Breast cancer
EWS: Ewing sarcoma
ICA: Independent component analysis
MF: Matrix factorization
NB: Neuroblastoma
NGS: Next-generation sequencing
NMF: Non-negative matrix factorization
PCA: Principal component analysis
RMS: Rhabdomyosarcoma
SRBCT: Small round blue cell tumors
TCGA: The cancer genome atlas network
