# Supplementary material for: DeepMF: deciphering the latent patterns in omics profiles with a deep learning method
Source: BMC Bioinformatics. 2019 Dec 27;20(Suppl 23):648. doi: 10.1186/s12859-019-3291-6 (PMC6933662; doi:10.1186/s12859-019-3291-6)
Supplement: Supplementary file 2 — Additional file 2 DeepMF performance on 1000×600 synthetic matrices. DeepMF denoising, imputation, and factorization performance on 1000×600 synthetic matrices with different pattern. Inside each pattern, from left to right: raw matrix, 10% random dropout, 50% random dropout, 70% random dropout; from top to bottom: before DeepMF, and DeepMF. The horizontal line plot show the sample latent factors, the vertical line plot refer to feature latent factors. A Matrix with pattern A; B Matrix with pattern B; C The transpose matrix of pattern B. [file 12859_2019_3291_MOESM2_ESM.pdf]

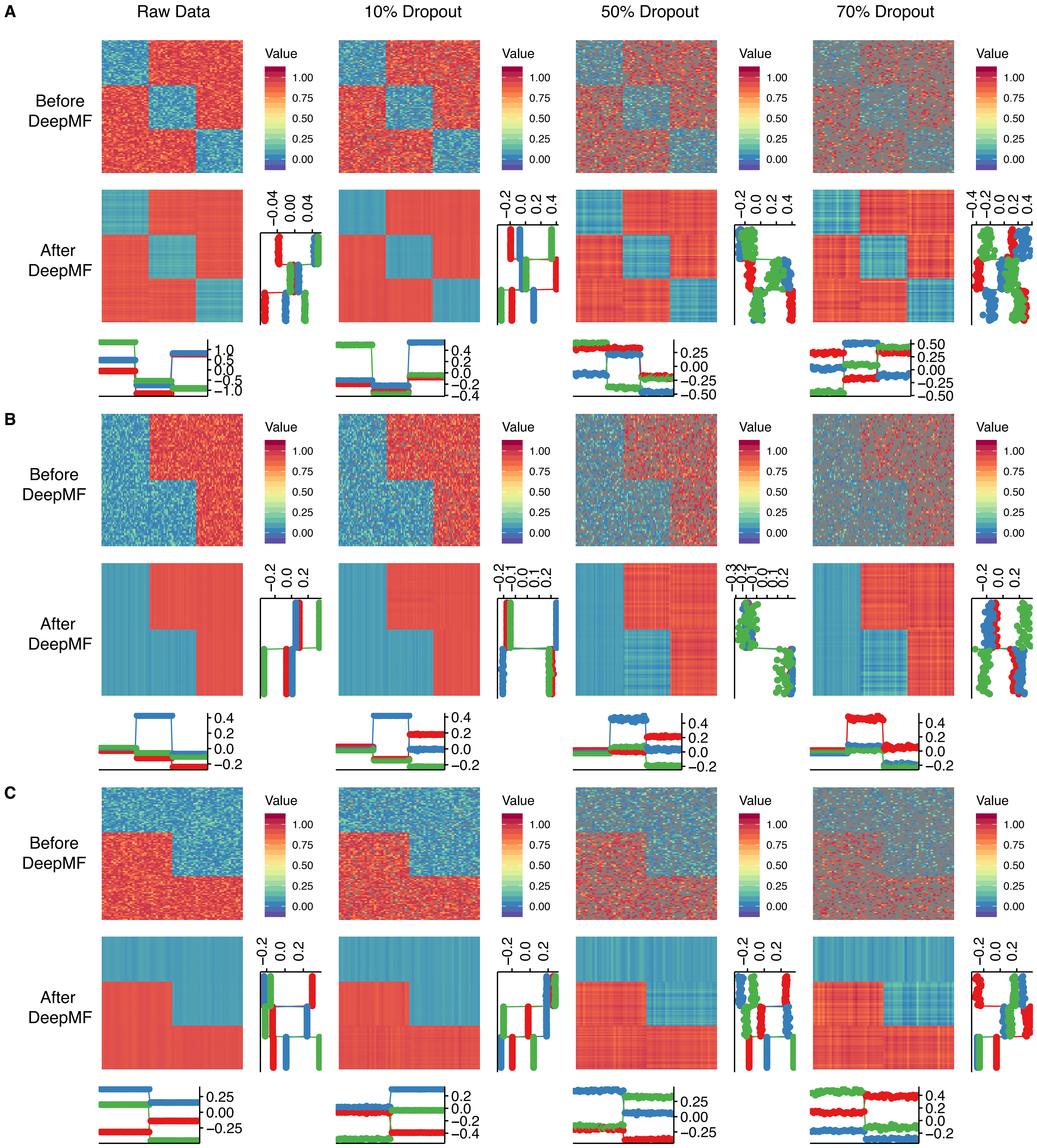
A
Raw Data
10% Dropout
50% Dropout
70% Dropout
Before DeepMF
After DeepMF
Value
1.00
0.75
0.50
0.25
0.00
B
C
