# Supplementary figures and images for: DeepMF: deciphering the latent patterns in omics profiles with a deep learning method
Source: BMC Bioinformatics. 2019 Dec 27;20(Suppl 23):648. doi: 10.1186/s12859-019-3291-6 (PMC6933662; doi:10.1186/s12859-019-3291-6)

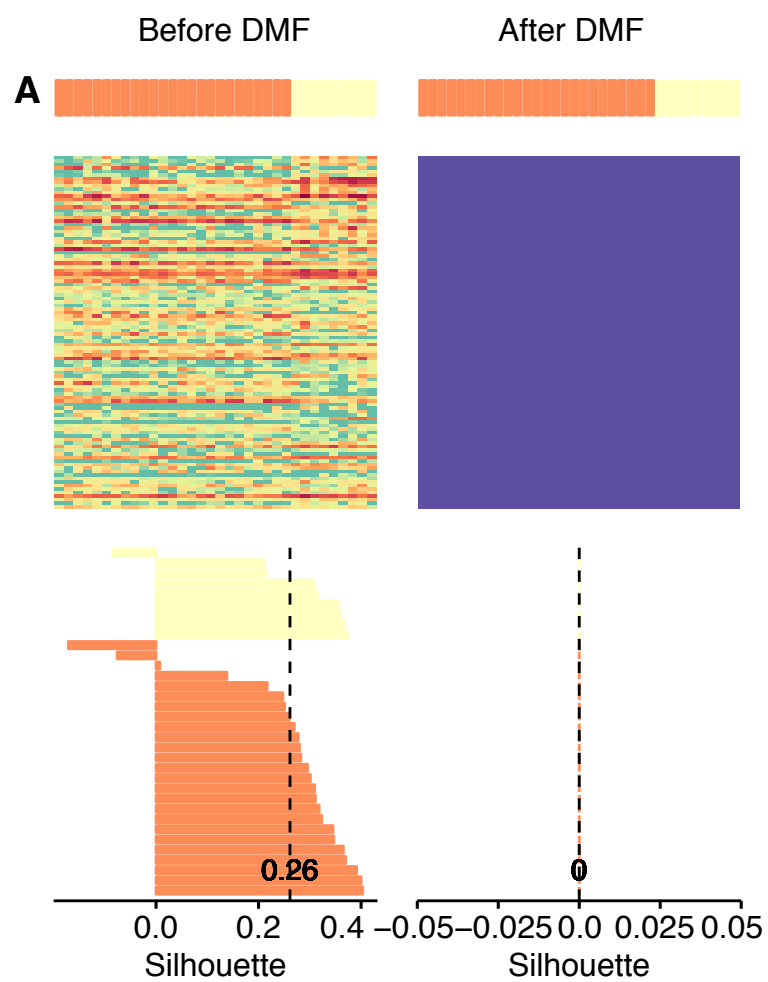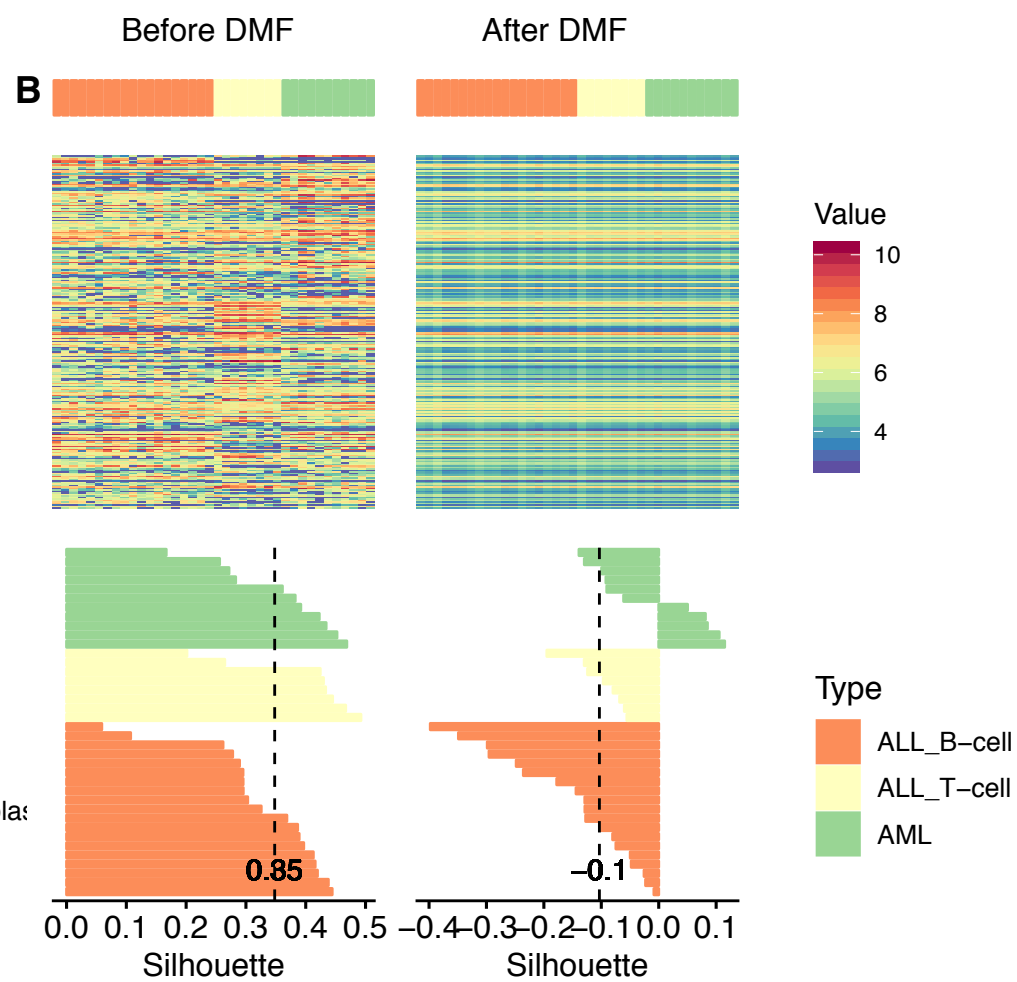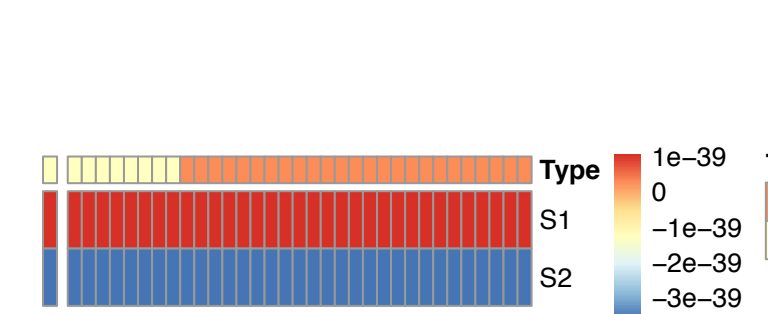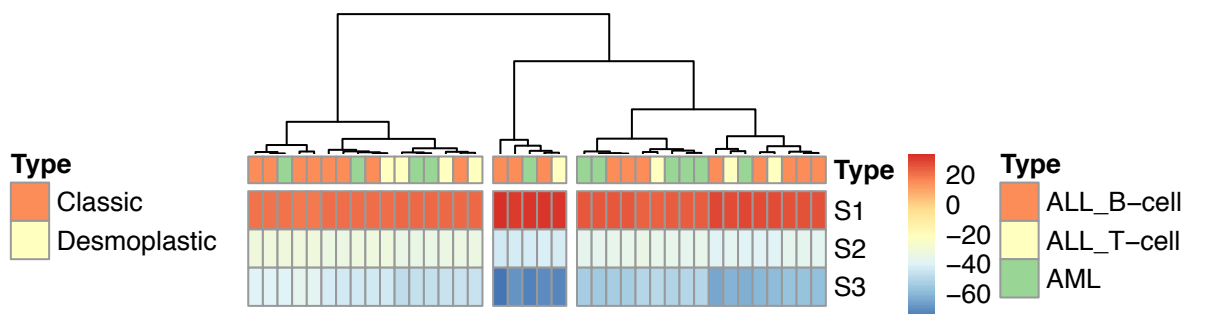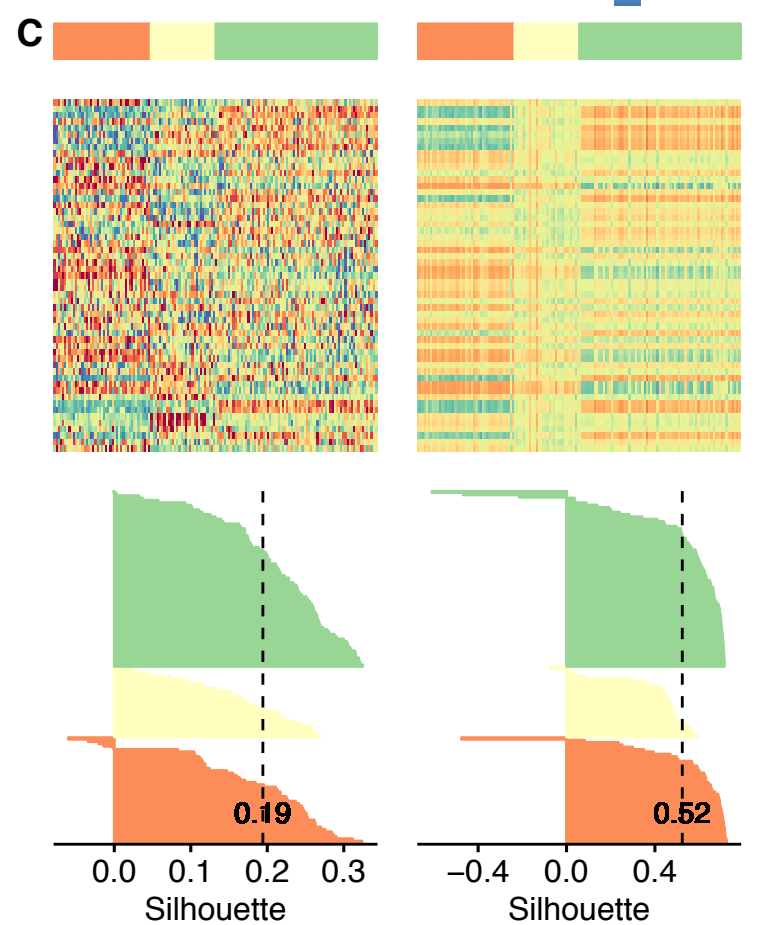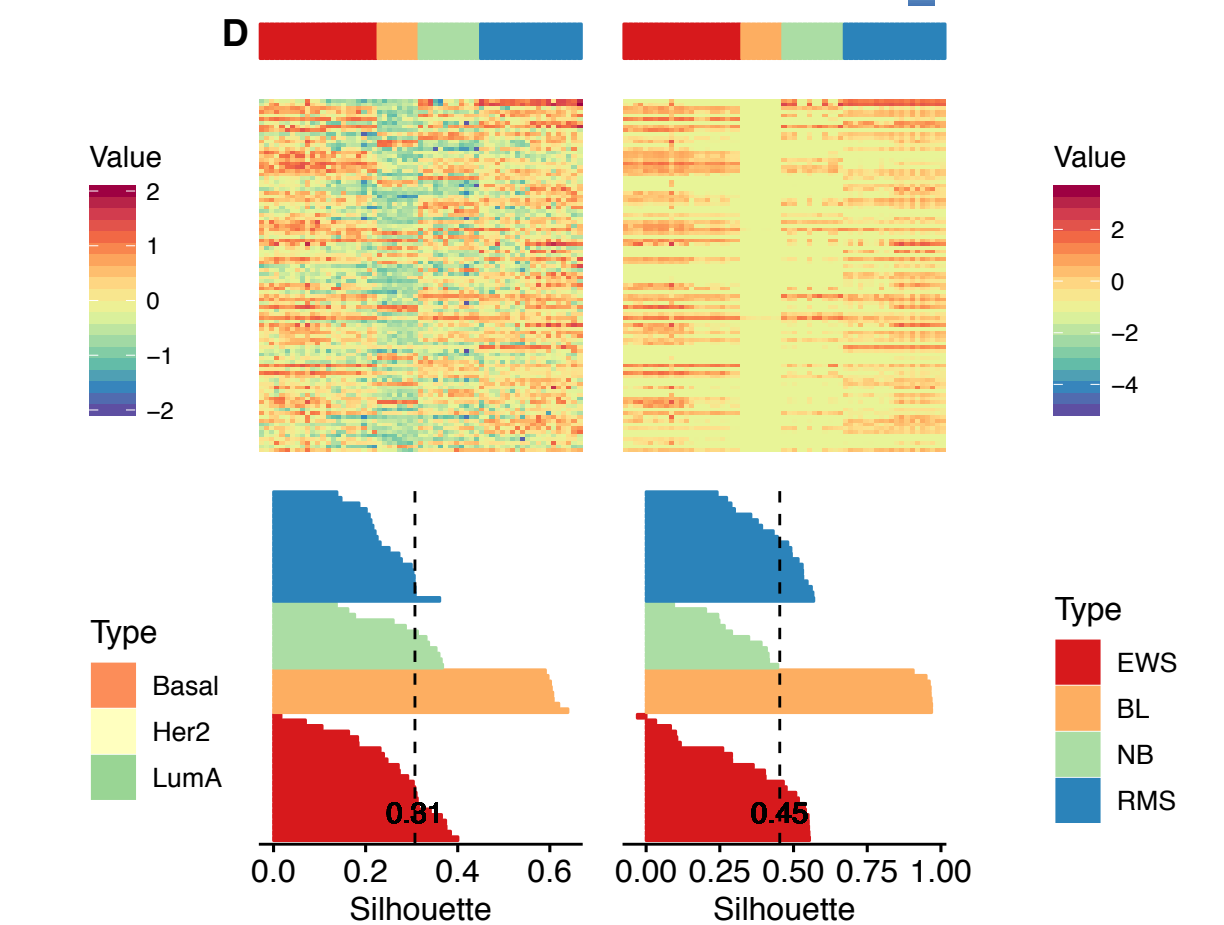

Supplement: Supplementary file 4 — Additional file 4 DMF denoising and factorization results on cancer data sets. A-D The heatmap presentation and Silhouette width of four cancer data sets. From left to right: matrix with before DMF, after DMF. The bottom: hierarchical clustering plots for sample latent matrice generated by DMF. A Medulloblastoma data set; B Leukemia data set; C TCGA BRCA data set; D SRBCT data set. [file 12859_2019_3291_MOESM4_ESM.pdf]

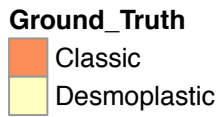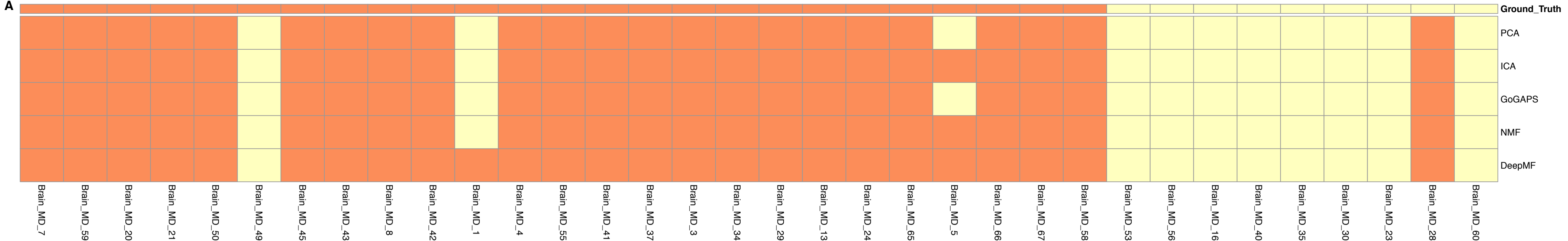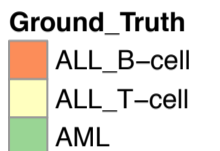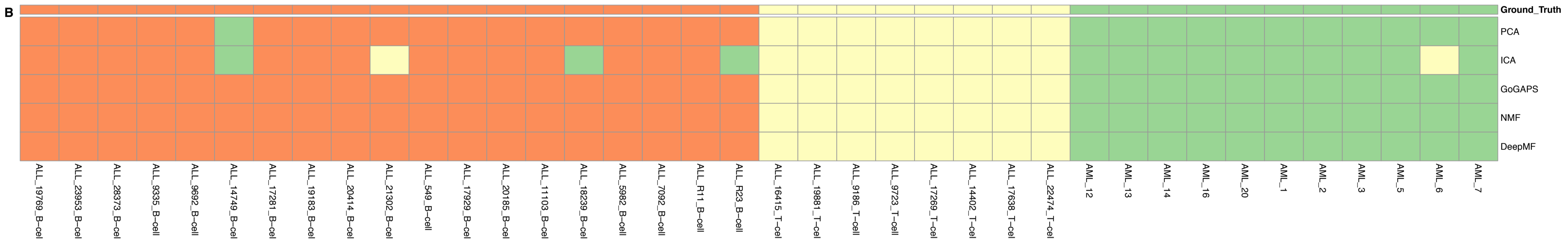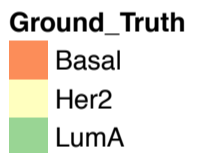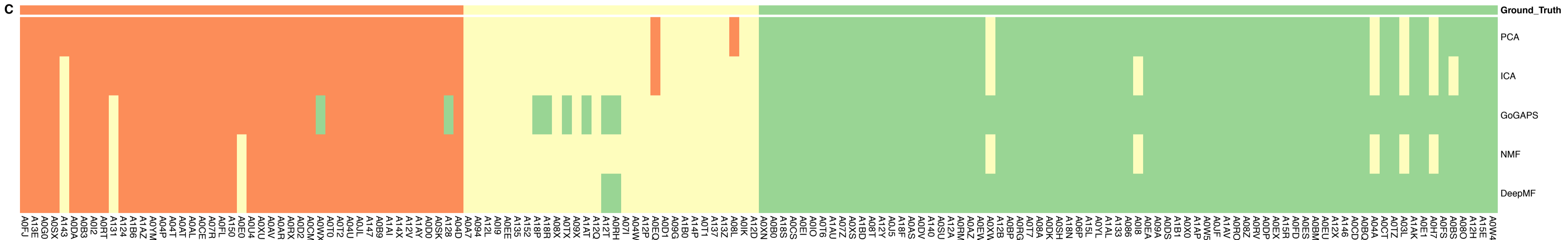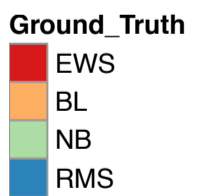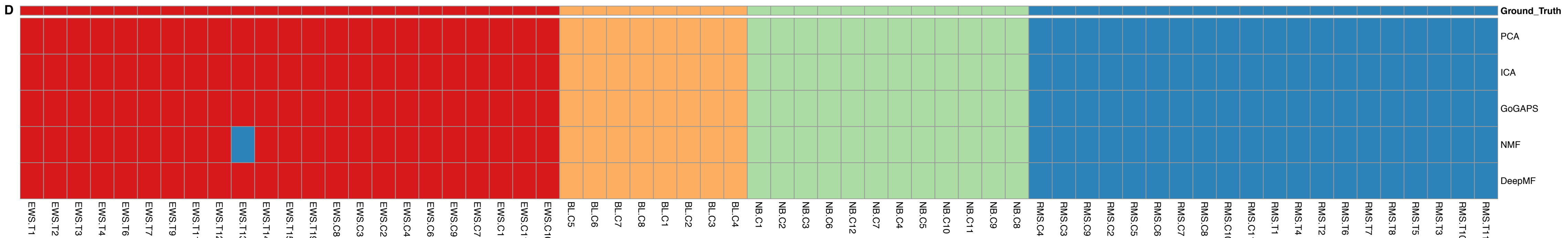

Supplement: Supplementary file 7 — Additional file 7 Hierarchical clustering results for sample latent matrices. The top row is the ground truth subtype label for each patients. The rest rows represent patient subtype assigned by PCA, ICA, CoGAPS, NMF, DeepMF, respectively. A Medulloblastoma data set; B Leukemia data set; C TCGA BRCA data set; D SRBCT data set. [file 12859_2019_3291_MOESM7_ESM.pdf]

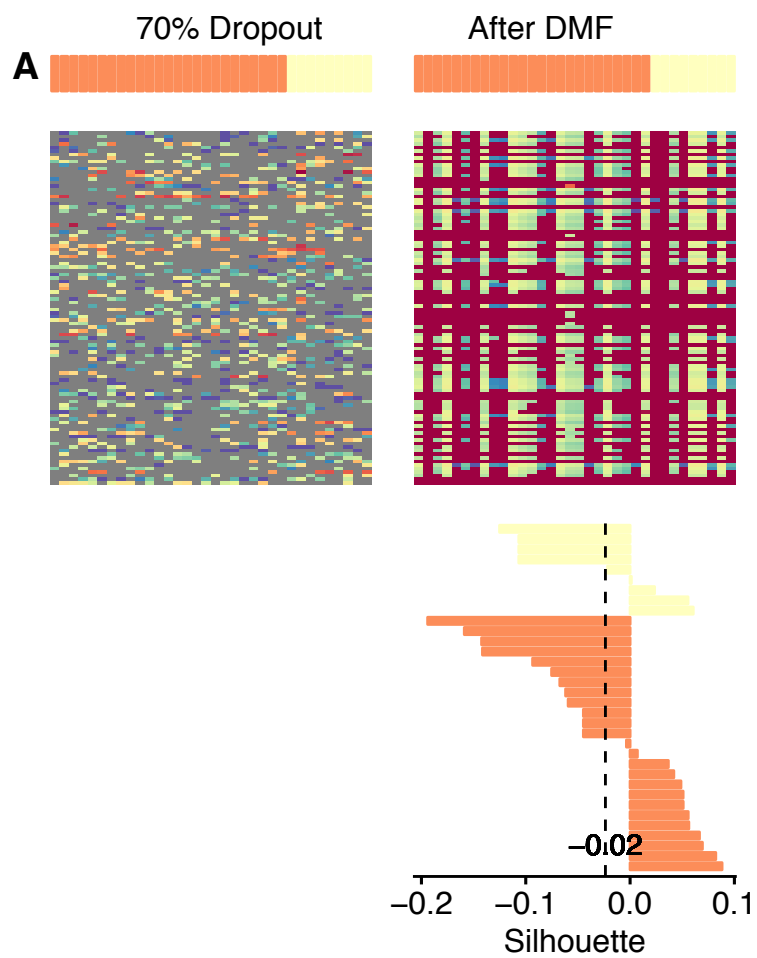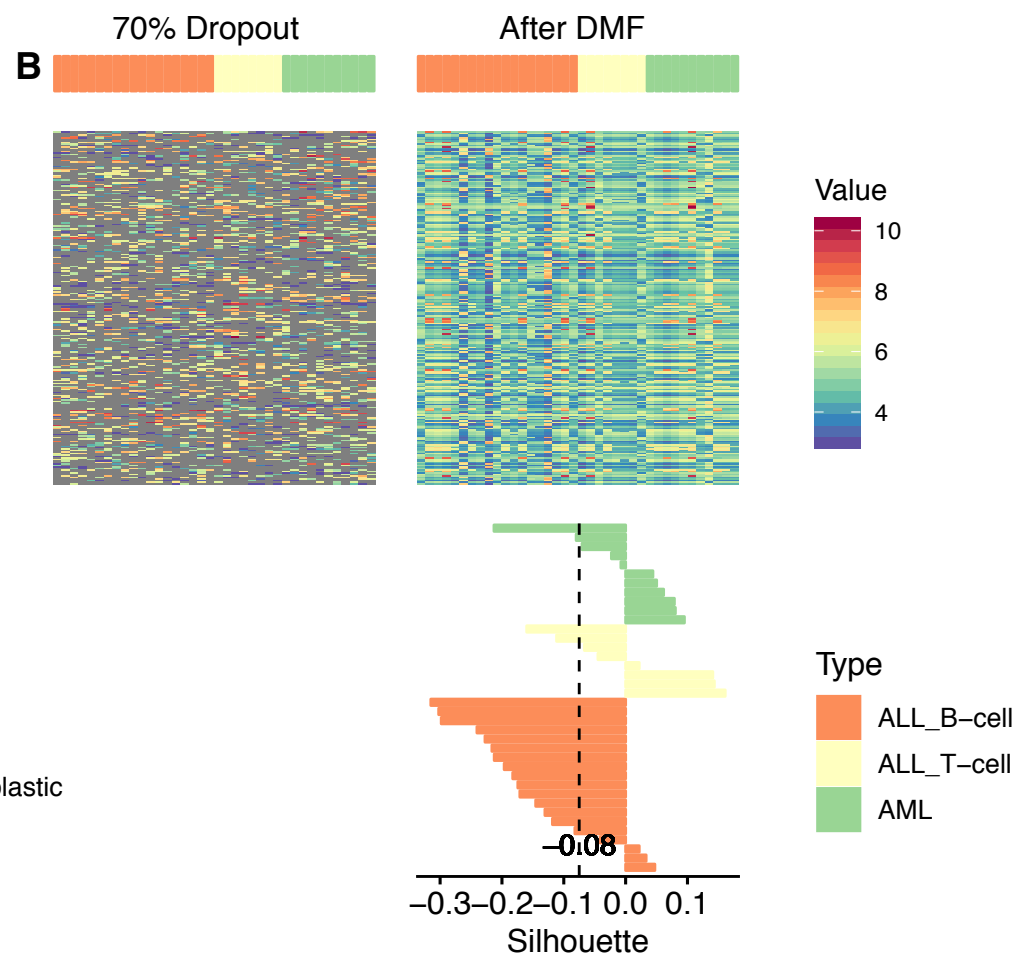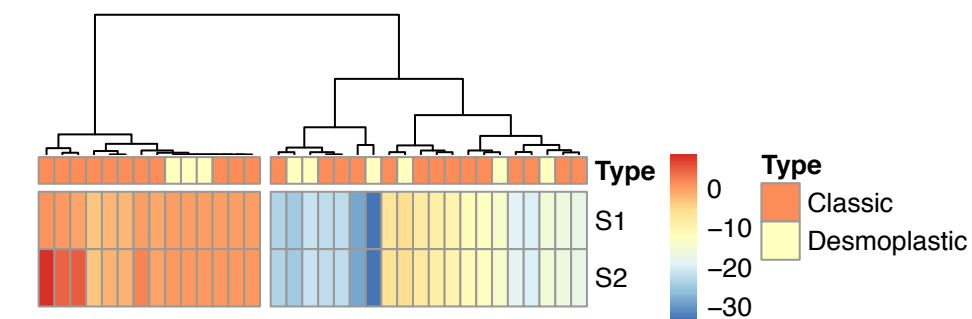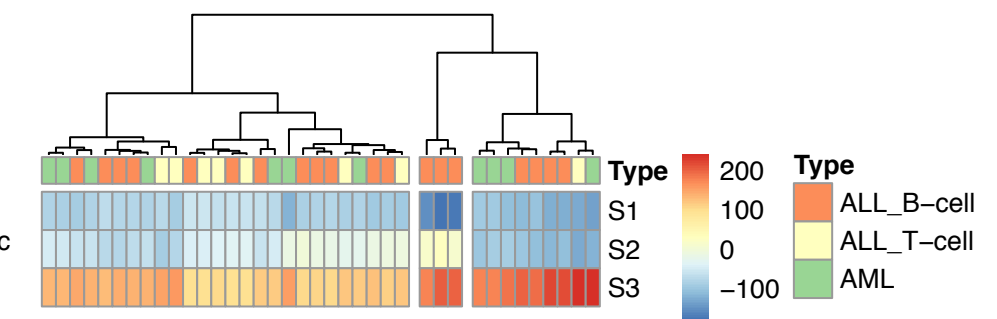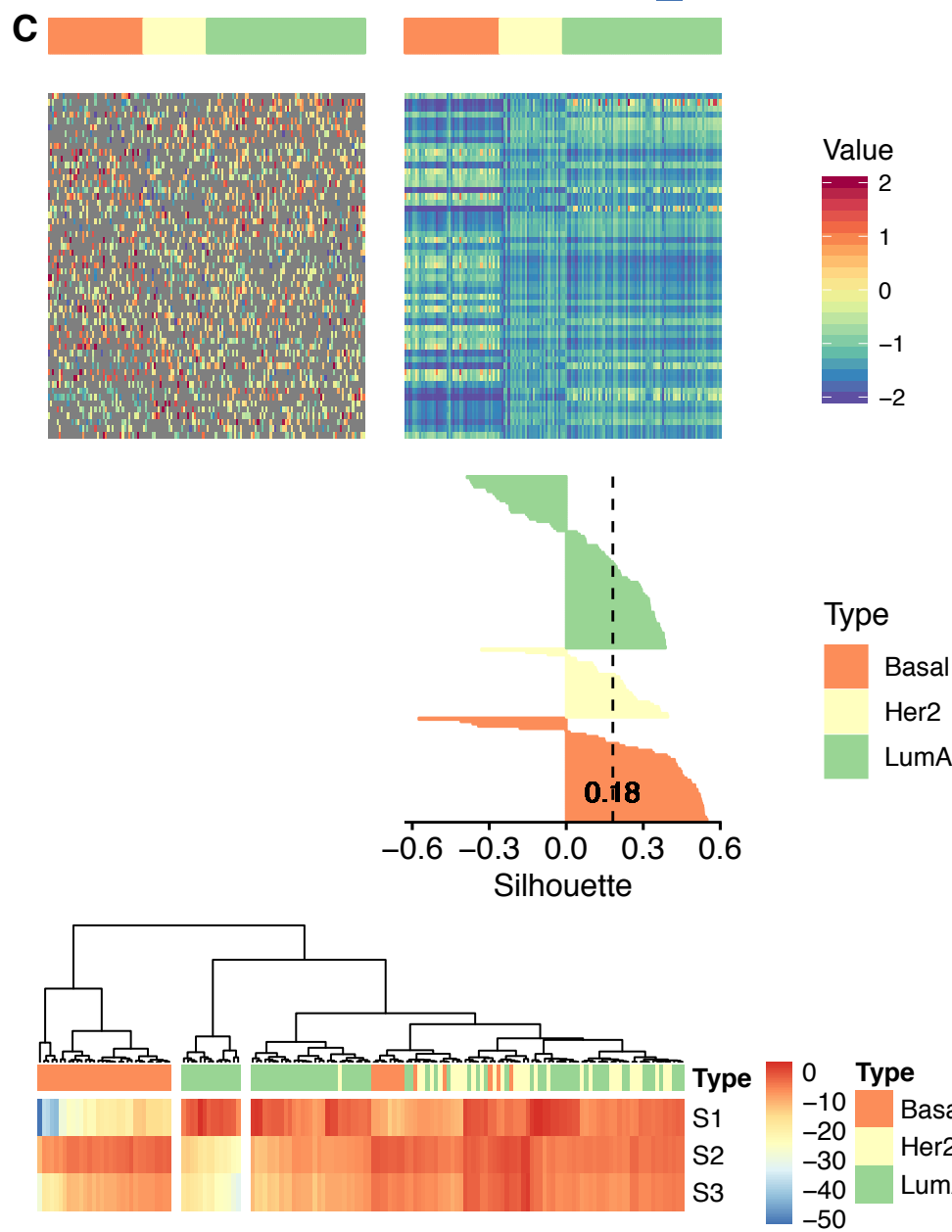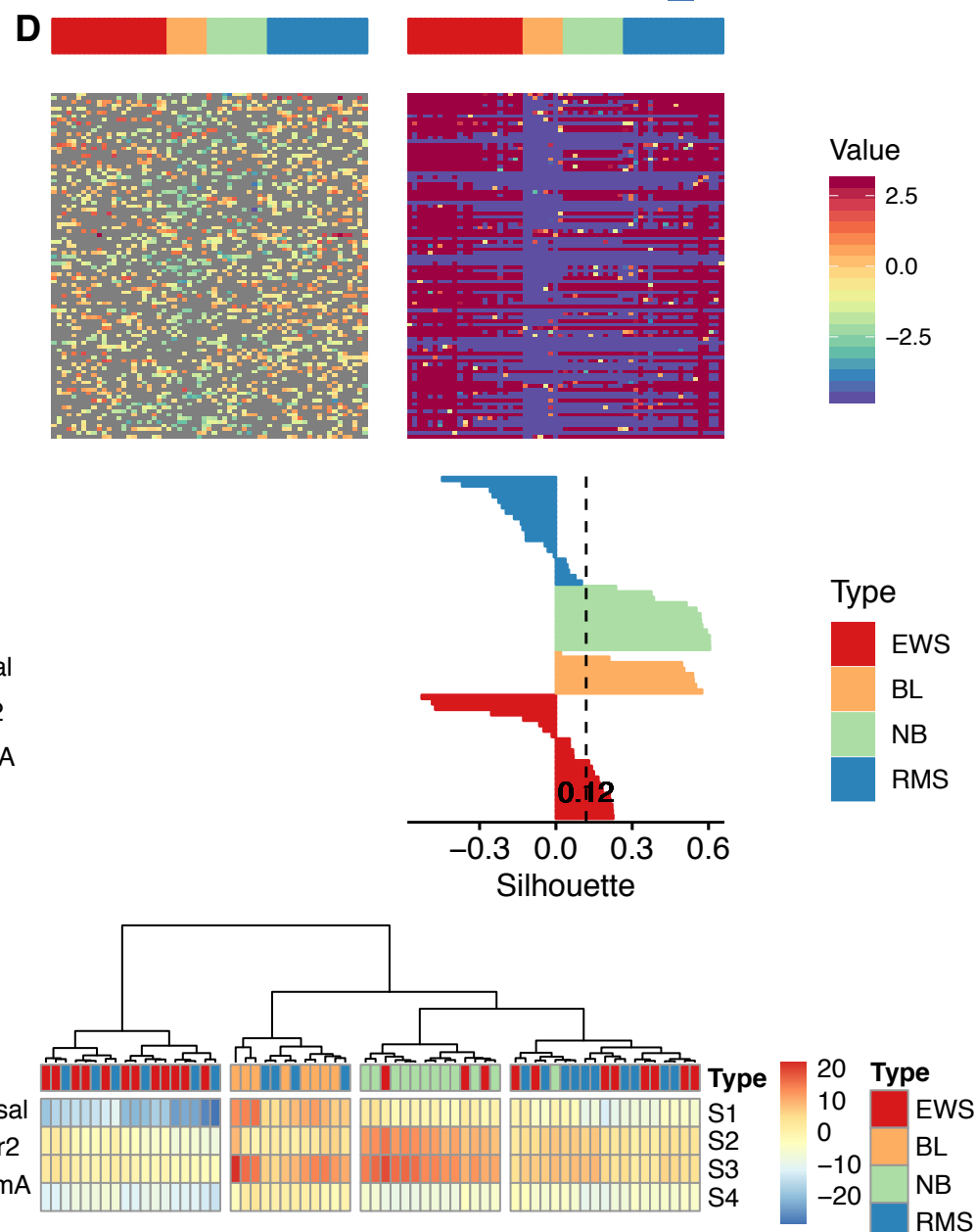

Supplement: Supplementary file 8 — Additional file 8 DMF imputation and factorization results on 70% sparse cancer data sets. A-D The heatmap presentation and Silhouette width of four cancer data sets with 70% random dropout. The gray tiles in heatmap indicate missing entries. From left to right: matrix with 70% random dropout, after DMF. The bottom: hierarchical clustering plots for sample latent matrice generated by DMF. A Medulloblastoma data set; B Leukemia data set; C TCGA BRCA data set; D SRBCT data set. [file 12859_2019_3291_MOESM8_ESM.pdf]

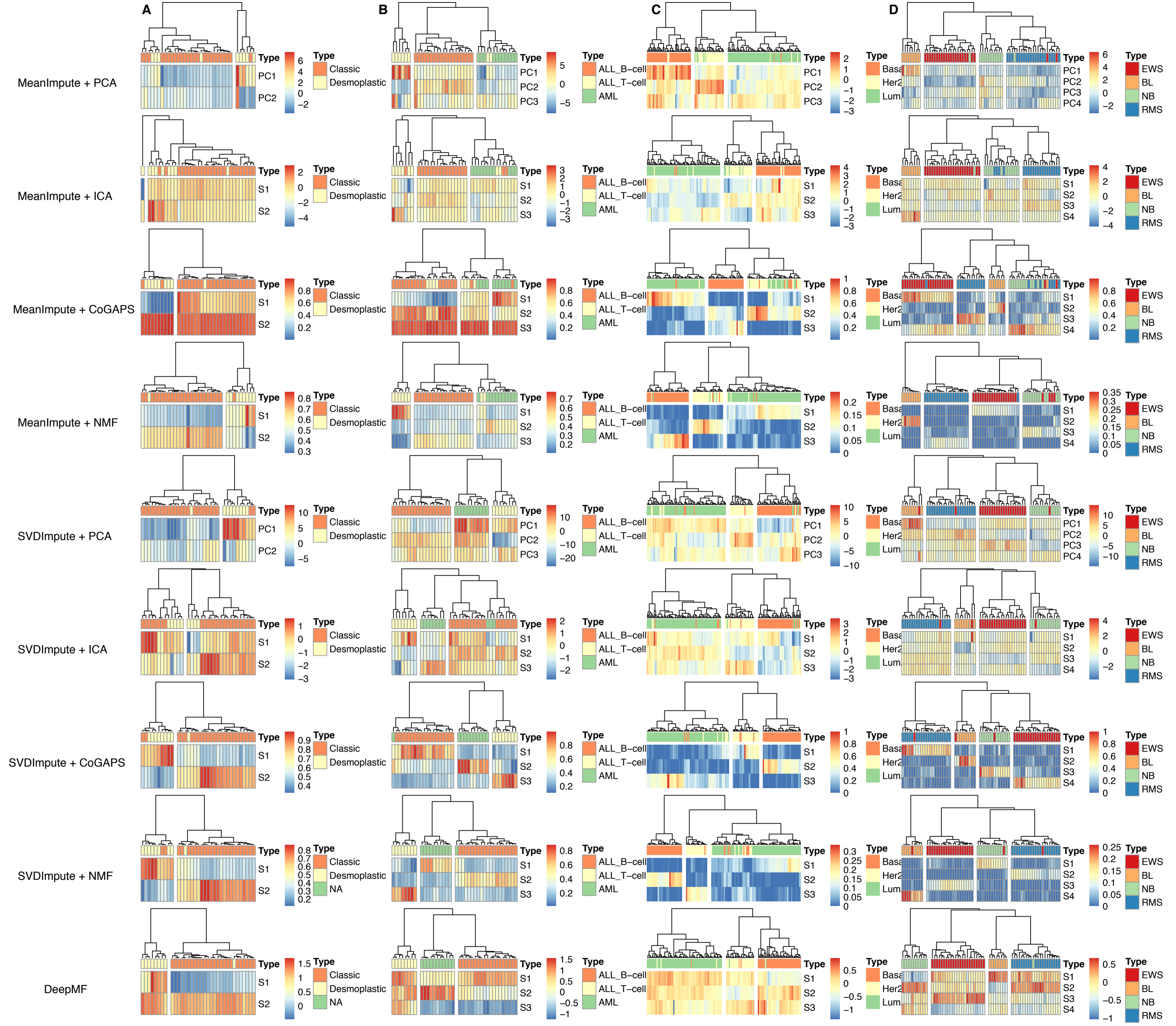

Supplement: Supplementary file 9 — Additional file 9 Hierarchical clustering plots for sample latent matrices generated from 70% random dropout data sets. Sample latent matrices are generated by two imputation tools and five matrix factorization tools on different cancer data sets with 70% random dropout. From top to bottom, each row represents sample latent matrices generated by meanImpute + PCA, meanImpute + ICA, meanImpute + CoGAPS, meanImpute + NMF, SVDImpute + PCA, SVDImpute + ICA, SVDImpute + CoGAPS, SVDImpute + NMF, DeepMF. A Medulloblastoma data set; B Leukemia data set; C TCGA BRCA data set; D SRBCT data set. [file 12859_2019_3291_MOESM9_ESM.pdf]

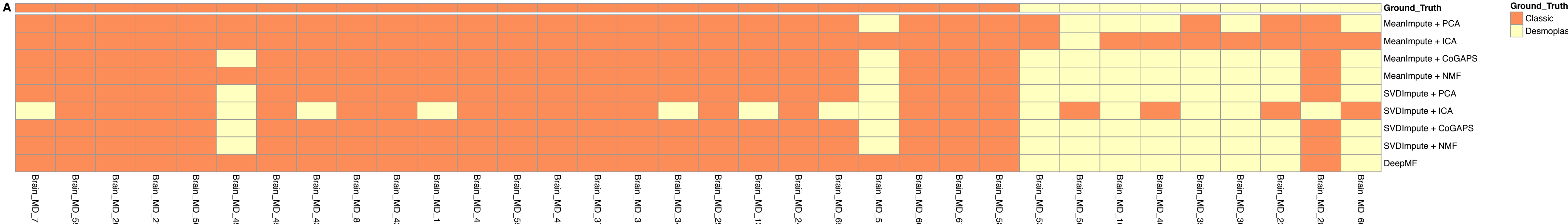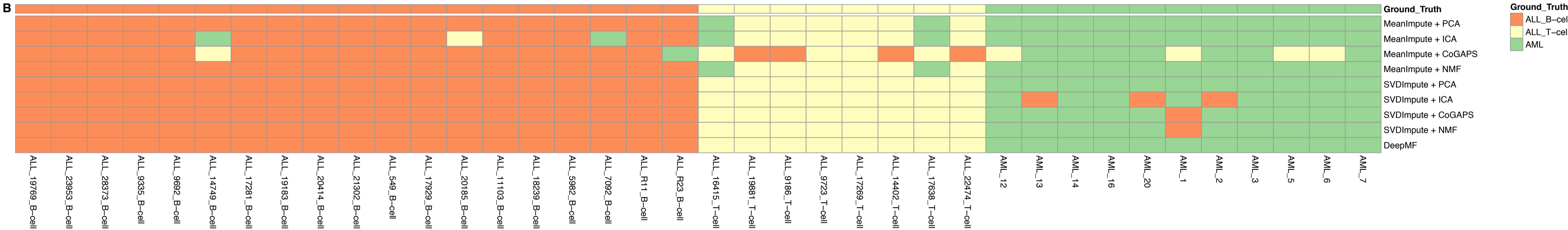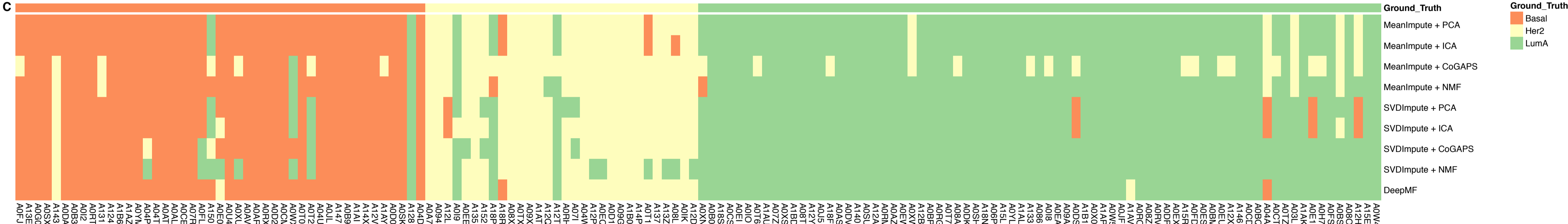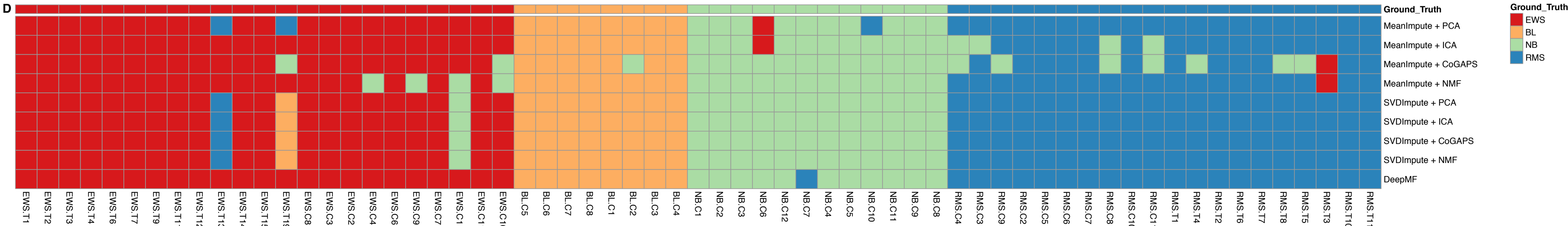

Supplement: Supplementary file 10 — Additional file 10 Hierarchical clustering results for sample latent matrices generated from 70% random dropout data sets. The top row is the ground truth subtype label for each patients. The rest rows represent patient subtype assigned by meanImpute + PCA, meanImpute + ICA, meanImpute + CoGAPS, meanImpute + NMF, SVDImpute + PCA, SVDImpute + ICA, SVDImpute + CoGAPS, SVDImpute + NMF, DeepMF, respectively. A Medulloblastoma data set; B Leukemia data set; C TCGA BRCA data set; D SRBCT data set. [file 12859_2019_3291_MOESM10_ESM.pdf]
