# Supplementary material for: DeepMF: deciphering the latent patterns in omics profiles with a deep learning method
Source: BMC Bioinformatics. 2019 Dec 27;20(Suppl 23):648. doi: 10.1186/s12859-019-3291-6 (PMC6933662; doi:10.1186/s12859-019-3291-6)
Supplement: Supplementary file 6 — Additional file 6 Hierarchical clustering plots for sample latent matrices. Sample latent matrices are generated by five matrix factorization tools on different cancer data sets. From top to bottom, each row represents sample latent matrices generated by PCA, ICA, CoGAPS, NMF, DeepMF. A Medulloblastoma data set; B Leukemia data set; C TCGA BRCA data set; D SRBCT data set. [file 12859_2019_3291_MOESM6_ESM.pdf]

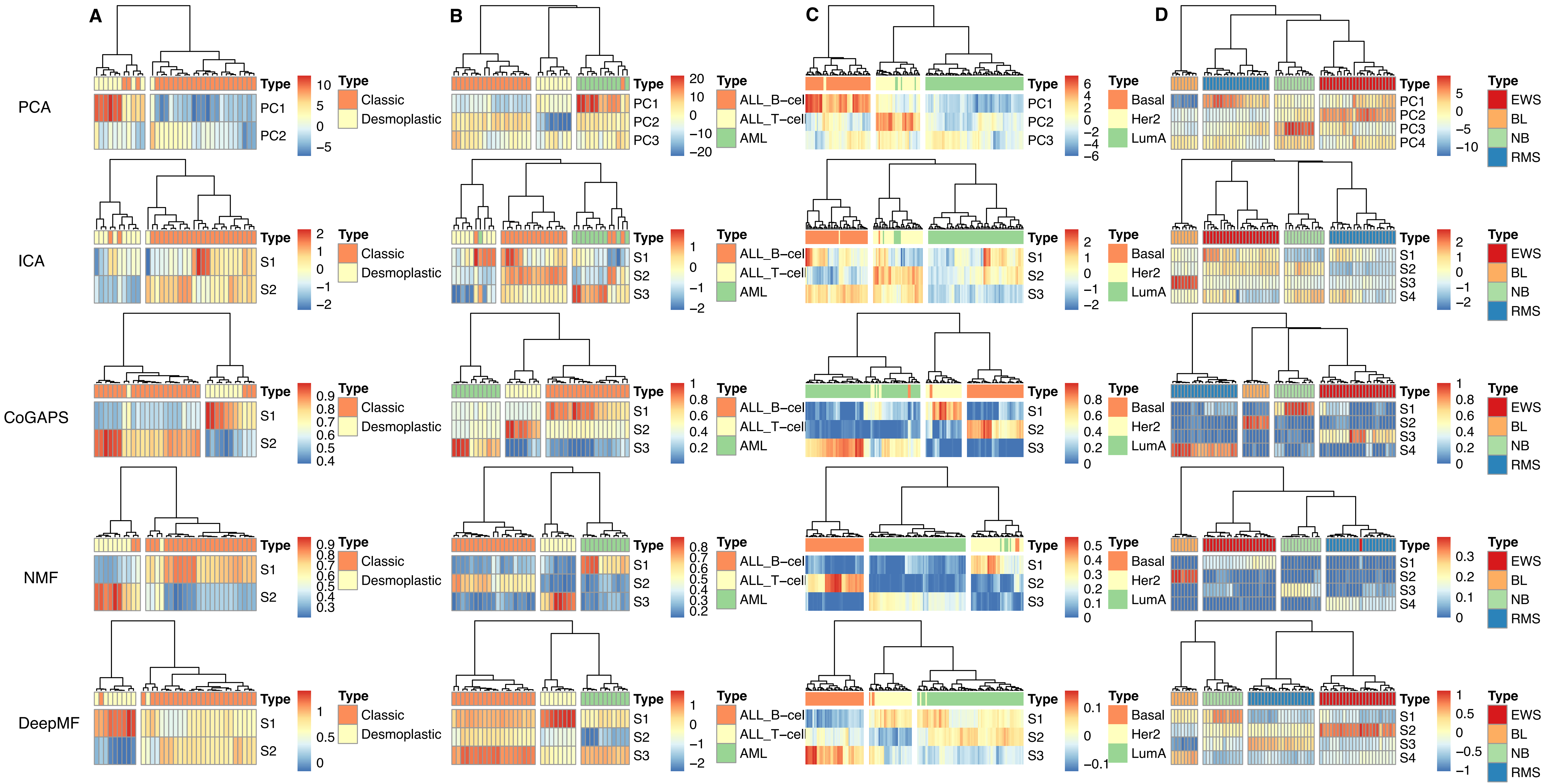

A
B
C
D
PCA
ICA
CoGAPS
NMF
DeepMF
Type
Classic
Desmoplastic
ALL_B−cel
ALL_T−cel
AML
Basal
Her2
LumA
EWS
BL
NB
RMS
PC1
PC2
PC3
PC4
S1
S2
S3
S4
